# Association of Drug Burden Index with grip strength, timed up and go and Barthel index activities of daily living in older adults with intellectual disabilities: an observational cross-sectional study

**DOI:** 10.1186/s12877-019-1190-3

**Published:** 2019-06-24

**Authors:** Juliette O’Connell, Martin C. Henman, Éilish Burke, Clare Donegan, Philip McCallion, Mary McCarron, Máire O’Dwyer

**Affiliations:** 1 School of Pharmacy and Pharmaceutical Sciences and IDS-TILDA, School of Nursing and Midwifery, Trinity College, Dublin, Ireland; 2 School of Pharmacy and Pharmaceutical Sciences, Trinity College, Dublin, Ireland; 3 IDS-TILDA, School of Nursing and Midwifery, Trinity College, Dublin, Ireland; 4 College of Public Health, Temple University, Philadelphia, USA; 5 Dean of Faculty of Health Sciences, Trinity College, Dublin, Ireland

**Keywords:** Ageing, Intellectual disability, Drug burden index, Anticholinergic, Sedative, Medication, Physical function, Grip strength, Timed up and go, Barthel index

## Abstract

**Background:**

Drug Burden Index (DBI), a measure of exposure to medications with anticholinergic and sedative activity, has been associated with poorer physical function in older adults in the general population. While extensive study has been conducted on associations between DBI and physical function in older adults in the general population, little is known about associations in older adults with intellectual disabilities (ID). This is the first study which aims to examine the association between DBI score and its two sub-scores, anticholinergic and sedative burden, with two objective measures of physical performance, grip strength and timed up and go, and a measure of dependency, Barthel Index activities of daily living, in older adults with ID.

**Methods:**

Data from Wave 2 (2013/2014) of the Intellectual Disability Supplement to the Irish Longitudinal Study on Ageing (IDS-TILDA) was analysed. Analysis of Covariance (ANCOVA) was used to detect associations and produce adjusted means for the physical function and dependency measures with respect to categorical DBI scores and the anticholinergic and sedative sub-scores (DBA and DBS).

**Results:**

After adjusting for confounders (age, level of ID, history of falls, comorbidities and number of non-DBI medications, Down syndrome (grip strength only) and gender (timed up and go and Barthel Index)), neither grip strength nor timed up and go were significantly associated with DBI, DBA or DBS score > 0 (*p* > 0.05). Higher dependency in Barthel Index was associated with DBS exposure (*p* < 0.001).

**Conclusions:**

DBI, DBA or DBS scores were not significantly associated with grip strength or timed up and go. This could be as a result of established limitations in physical function in this cohort, long-term exposure to these types of medications or lifelong sedentary lifestyles. Higher dependency in Barthel Index activities of daily living was associated with sedative drug burden, which is an area which can be examined further for review.

**Electronic supplementary material:**

The online version of this article (10.1186/s12877-019-1190-3) contains supplementary material, which is available to authorized users.

## Background

Intellectual disability (ID) is a neurodevelopmental disorder which manifests early in life and is characterised by impairments of general mental abilities that affect adaptive functioning [1]. Historically, people with ID had greatly reduced life expectancy compared to those without ID [2]; however, in recent years the life expectancy among this group has increased and people with ID are living into middle and old age [2, 3]. This success is derived from numerous factors, including better access to medical intervention and improved health status [4]. However, people with ID still experience premature mortality. The Confidential Inquiry into premature deaths of people with ID, commissioned by the Department of Health in England, identified that there was a higher probability of preventable deaths of people with ID, owing to untreated health problems and deficiencies in healthcare provision for this population [5]. Age-related changes tend to occur earlier in the lives of people with ID, including those with Down syndrome, cerebral palsy, Cornelia de Lange syndrome, Prader-Willi syndrome and fragile X syndrome [2]. These age-related changes can include early menopause (Down syndrome; fragile X syndrome), Alzheimer’s disease (Down syndrome), degenerative arthritis and faster musculoskeletal system ageing (cerebral palsy) and early development of osteoporosis and premature greying of hair (Cornelia de Lange syndrome) [2, 6].

It has been reported that seven out of ten adults with ID over 40 years old experience multimorbidity, with mental illness, neurological disease, gastrointestinal disease and eye disease among the most prevalent conditions [7]. Higher rates of cardiac abnormalities, musculoskeletal disorders, hypothyroidism, early menopause, epilepsy, dementia and hearing and visual impairments are observed in adults with ID as they age compared to the general population [8]. Older adults with ID face different challenges as compared to older adults without ID. The trend of deinstitutionalisation for adults with ID, while important to enabling better quality of life and social connectedness, may also result in reduced access to services required to meet the needs of adults ageing with an ID. Health practitioners in primary care may fail to identify special and unique problems experienced by people with ID who are ageing [8]. While people with ID may typically be treated by the same General Practitioner (GP) throughout their lifetime, it has been well-documented that disparities in healthcare exist [5, 9, 10]. As a result of multiple morbidities and the higher prevalence of certain medical conditions in this population, including epilepsy and mental health conditions [7, 11], which frequently require accessing more specialist services such as neurology and psychiatry, problems with coordination of care and information sharing are relatively common for people with ID as they may access primary care for some treatments and secondary care for others [5, 9], and have been identified as factors which contribute to premature death [5]. Other issues around care of people with ID such as difficulty communicating, diagnostic overshadowing and atypical presentation of certain illnesses can make diagnosis and treatment of health conditions in this population more difficult and as a result lead to disparities in healthcare [9, 10].

In the absence of appropriate alternative interventions, older adults with ID may have increased risk of experience of high levels of exposure to multiple medicines, including those with anticholinergic and sedative effects [12, 13]. Older adults with ID are more likely to receive medications with anticholinergic properties, intermediate and long-acting benzodiazepines and antipsychotics than older adults in the general population [14]. A cross-sectional study of ageing in people with ID in Ireland identified 70.9% were exposed to medications with anticholinergic effects as measured by the Anticholinergic Cognitive Burden Scale (ACB) [12]. Anticholinergic and sedative burden as measured by the Drug Burden Index (DBI) has been found to be higher among older people with ID than those without ID – 78.6% of older adults with ID were regularly exposed to medications with anticholinergic and sedative effects [15], compared to 28–49% among older adults without ID, depending on study setting and population [16–32]. These types of medications have well-documented adverse effects on older adults in the general population, such as falls, frailty, fracture liability, and physical and cognitive impairment [33–41] and, as a result, studying anticholinergic and sedative medication use in older adults with ID was identified as an area of research interest. Studies of the adverse effects of these types of medication on older adults with ID are limited, though existing research has found that use of anticholinergic medications in older adults with ID is associated with daytime drowsiness and chronic constipation [12].

The DBI has been developed as a tool to measure the total exposure of an individual to medications with anticholinergic and sedative activity [21]. The DBI is a dose-related measure which assesses quantitatively the burden an individual is exposed to from medications with these effects. The inventory of medications is typically selected by literature review and medication analysis to assign medications as having anticholinergic and/or sedative effects [15]. The total daily dose of each medication with anticholinergic/sedative activity that a person is exposed to is examined with respect to the minimum effective daily dose for that medication. The minimum effective daily dose is selected in order to approximate the DR_50_, or dose required achieve 50% of maximal contributory effect at steady state [42]. An individual exposed to the minimum effective daily dose of a DBI medication will be assigned a score of 0.5 for that medication. This calculation is performed for each relevant medication, and scores are summed to give a cumulative DBI score for the individual. Existing literature uses a common categorisation of scores: DBI score 0 (no DBI exposure), DBI score 0 > 1 (low) and DBI score ≥ 1 (high). [16, 17, 19–23, 31, 43, 44]. It has been internationally validated and has been associated with impairment of physical function in older people without ID, including poorer performance in measures such as walking speed, balance, grip strength, timed up and go (TUG) and the Short Physical Performance Battery [18–20, 45]. DBI has also been found to be associated with a greater risk of transitioning from the robust state to the pre-frail state in community-dwelling men aged 70 and older [46]. DBI has recently been analysed in older adults with ID, and it has been found that DBI scores are much higher in this population than those reported in older adults without ID, with 54% older adults with ID having a high DBI score (≥1) compared to between 5 and 29% of older adults without ID [15]. In particular, exposure to medications with anticholinergic effects is much more frequent in older adults with ID [12, 15]. In addition, high DBI score has been found to be significantly associated with increased dependency in Barthel Index (BI) activities of daily living in older adults with ID [15].

Physical performance is an important marker of functional independence in older adults [45]. Physical fitness measurements have only in recent years been assessed in older adults with ID. Hilgenkamp et al. [47, 48] have examined the feasibility and reliability of physical function measures in this group and concluded that grip strength is an appropriate method of measuring function in this group. Enkelaar et al. (2013) [49] have identified that TUG is a feasible method of assessing balance and gait capacities in older adults with ID.

The association between performance in physical function measures and DBI has been examined in a number of studies of older adults without ID. However, there is a lack of research in the area of the association of drug burden on physical function in older adults with ID. To our knowledge, this is the first study to examine physical function measurements in relation to DBI in older adults with ID.

### Aim

The aim of this study is to examine the association between Drug Burden Index and performance in two physical function measures, grip strength and TUG, and a measure of dependency, Barthel Index, in a cohort of older adults with ID at a cross-sectional level to establish if similar exposure in people with ID leads to the same functional effects as has been reported in the general population.

More specifically, the objectives were to identify associations and adjusted means for:

- Grip strength
- Timed up and go
- Barthel Index

with respect to exposure to DBI medications, DBI medications with anticholinergic activity (DBA) and DBI medications with sedative activity (DBS) and three-level, dose-related categorical DBI, DBA and DBS scores.

## Methods

### Design

Data for this study was obtained from older adults with ID enrolled in Wave 2 (2013/2014) of the Intellectual Disability Supplement to the Irish Longitudinal Study on Ageing (IDS-TILDA), an observational, cross-sectional study, and has been described in detail elsewhere [15]. IDS-TILDA is a large scale, nationally representative longitudinal study which examines the ageing of people with ID [50, 51].

### Participants

The sampling frame for Wave 1 of this study was the National Intellectual Disability Database (NIDD). NIDD collects information to define the specific health services in use or required by people with ID in the Republic of Ireland. Staff at NIDD randomly selected 1800 personal identification numbers (PINs) and an invitation pack with a consent form was sent to each potential participant. Participants signed written informed consent independently or, if this was not possible, a family member/guardian signed a letter of agreement for their family member to participate. At Wave 1 of the study (2010/2011), 753 people aged ≥40 years participated. Participants were aged 40 years or older to account for the reduced life expectancy and presentation of older age conditions, e.g. dementia, at a younger age in people with ID [2]. Ethical approval for the study was granted by the Faculty of Health Sciences Research Ethics Committee in Trinity College Dublin. In addition, local and/or regional ethical committee approval was granted from each service provider (*n* = 138).

**Fig. 1:**
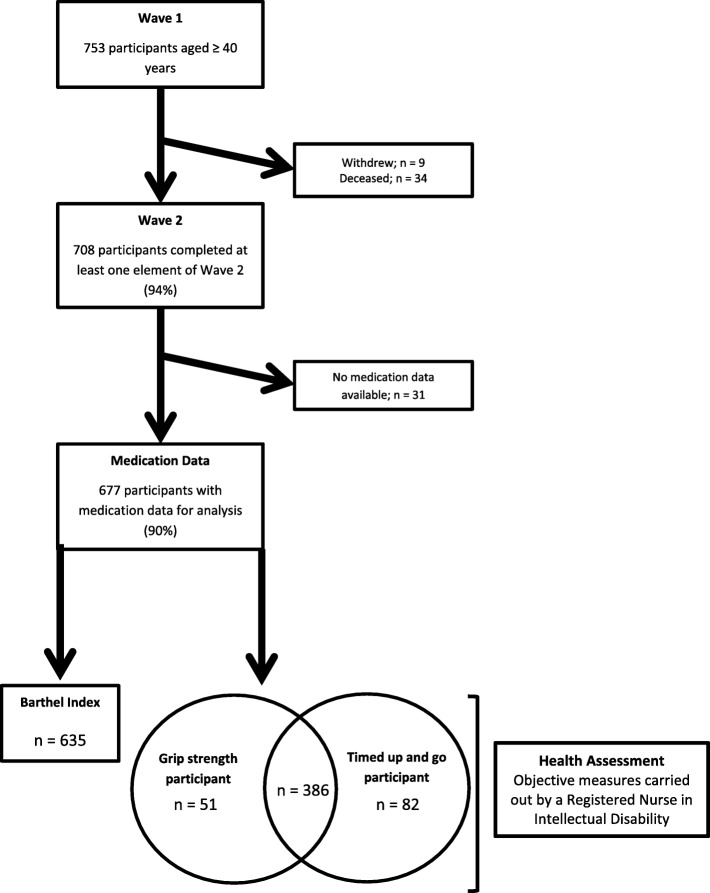
Flow Chart for IDS-TILDA

All living Wave 1 participants (*n* = 719) were invited to participate in Wave 2. The study population with available medication data was 677 (95.6%) (Fig. 1).

Data was gathered by three means.

- Firstly, participants were provided with a pre-interview questionnaire (PIQ) 1 week in advance of the face-to-face interview. The PIQ captured data in a number of areas, including physical health and medication data. In the majority of cases (92.8%; *n* = 628), the PIQ was completed by proxy (a key worker or family member known to participants for at least 6 months). It was provided in advance of the interview in order to allow adequate time for completion, which involved accessing medical records.
- Secondly, a computer assisted personal interview (CAPI) was used by field researchers in the face-to-face interview with participants. Three different interview techniques were employed to facilitate the needs of participants – (1) participants completed interview independently, (2) participants completed interview with assistance from a proxy or (3) interview with a proxy only on behalf of the participant. The PIQ and CAPI for Wave 2 of IDS-TILDA are available online [52].
- Thirdly, a registered nurse in intellectual disability (RNID) carried out a health assessment which included the objective measures of physical function – grip strength and timed up and go (TUG). In addition, body mass index (BMI) and Lunar Achilles GE Quantitative Ultrasound (QUS) were assessed in order to collect data on overweight/obesity and osteoporosis/osteopenia [50]. Adaptable and accessible materials and methods were developed to assist participants who partook in the health assessment. This component has been described in detail elsewhere, for further details see Burke et al., 2014, 2016a & 2016b [50, 53, 54].

Figure 1 displays the flow chart of the study.

The STROBE (Strengthening the Reporting of Observational Studies in Epidemiology) reporting guidelines for cross-sectional studies were used [55, 56].

### Drug burden index

Medication data was collected by asking participants/proxies to complete the medication section of the Pre-Interview Questionnaire (PIQ). Participants/proxies were asked “Can you tell me what medications (including prescribed or over the counter (OTC)) and supplements you take on a regular basis (like every day or every week)?”

Medication data was recorded by brand name/International Non-Proprietary Name (INN), dose, frequency, route of administration and date on which medicine was initiated in the PIQ.

The Anatomical Therapeutic Chemical Classification System (ATC) was used to code medications and data was verified by two pharmacists. Medications which were topical, inhaled, “as required” or recorded for use which was not regular (eg pre-dental/medical procedures; acute seizure control) were excluded from analysis. The only exception was atropine eye drops (ATC code S01FA01), which are considered to have clinically significant systemic effects [57]. Prochlorperazine was recoded from ATC code N05AB04 (Antipsychotics) to A04A (Antiemetics and Antinauseants) as the dosages reported in the IDS-TILDA population fell within the dosage range used for treatment of Meniere’s syndrome, nausea and vomiting (10 – 40 mg daily) as opposed to schizophrenia and other psychotic disorders (75 – 100 mg daily) [58].

The DBI score for each participant was calculated using the following formula:

$$ Drug\ Burden\ Index=\sum \frac{D}{\delta +D} $$

where D is the daily dose and δ is the Minimum Daily Dose (MDD). The MDD is used as an estimate for the DR_50_, the daily dose to achieve 50% of the maximum of anticholinergic and/or sedative effect. Medications were identified as having clinically significant anticholinergic and/or sedative effects by referring to relevant studies [12, 21, 34, 43, 59] and the Irish medicinal product literature (Summary of Product Characteristics, SmPC), available from the Health Products Regulatory Authority (HPRA) [60] Medications with both anticholinergic and sedative effects were classified primarily as anticholinergic as per previous studies. MDDs were identified as the lowest effective daily dose listed in the Irish medicinal product licenses from the HPRA [60]. This medication inventory and the DBI tool for this cohort has been described in more detail elsewhere [15].

### Physical function measures

Two measures of physical function were completed: grip strength and TUG. Grip strength is a measure of maximum voluntary force of the hand [61] and has been found to be a valid and reliable instrument for measuring hand strength [62]. Three pre-assessment skills processes were conducted by an RNID. Initially the person was asked to sign their consent and the researcher noted the hand used for signing. Then the person was asked which was their dominant hand (strongest or which one they used for doing most things). Dominance may be a challenging concept for people with ID to determine. If there remained a difficulty the researcher got the participant to squeeze two of her fingers on each hand and noted which side appeared stronger. Grip strength was measured using a Jamar Hydraulic Dynamometer (two measurements on each hand), a valid and reliable instrument for measuring hand strength [63]. Grip strength is reported as a continuous variable (in kg) and as a categorical variable, stratified by age and gender according to the manufacturer’s instructions [53]. Four grip strength readings were obtained, two from the right hand and two from the left, and all results were recorded. The grip strength measure was demonstrated by the RNID prior to the participant’s measurement. The participant was encouraged to squeeze as hard as they could for as long as they could or until the needle stopped rising. Once the needle stopped rising the participant was instructed to stop squeezing. Grip strength was measured with participant sitting, with forearms flat on the arms of a chair, feet flat on the ground, as per the protocol used for grip strength assessment (Roberts et al., 2011 [64]).

TUG was used as a means of assessing the proximal muscle strength, balance and executive function of participants [65]. A standard chair was placed against a wall to provide secure support. This chair measured 45 cm from the floor to the top of the armrest. A tape measure was used to measure a distance three meters from the chair and high visibility tape was used to mark this point. Participants were instructed to get up from a sitting position in the chair, walk to the marked point, turn around, walk back to the chair and sit down. The procedure was timed and recorded in seconds with a Seca stopwatch [53].

After assessing the safety of performing the TUG assessment and obtaining verbal consent, the RNID gave the following verbal directions to the participant:

“I am going to do a walking test. I will get you to sit in this chair with your back resting against the back of the chair. On the word GO you should stand up walk to the line on the floor, turn around, walk back to the chair and sit down. Please walk at your regular pace. Is that OK? Do you have any questions? I will demonstrate this now”.

After addressing any relevant questions or concerns, the RNID demonstrated performance of the test. If the RNID had concerns that the respondent did not fully understand the instructions, they allowed them to do a practice prior to the timed test.

Following this, the RNID gave this direction:

“I will now get you to do that. Do you have any questions before we begin? I am going to time you. You should walk at your usual pace. Are you ready? ‘Go’”

This procedure was consistent with that which was employed by Salb et al. (2015) for TUG, whereby participants were given a demonstration of the test by the investigator, offered a trial, invited to “walk in a comfortable and safe (e.g. unrushed) speed” and begin the test on the rater’s command “and go” [66].

Prior to assessment, participants were offered the opportunity to practice for the grip strength measurement by squeezing a rubber ball and were offered a practice run of the TUG.

### Barthel index

The Barthel Index measures the level of dependence of an individual in ten instrumental activities of daily living (mobility, using stairs, dressing, bathing, grooming, feeding, transfer, toileting and bladder and bowel continence). It consists of an ordinal scale with range 0–20 [67, 68]. A modified form of BI activities of daily living was created for this population (Additional file 1). Lower scores indicated poorer physical function.

As per a previous study, participants with two or more missing values were excluded from the Barthel Index evaluation (*n* = 42) [15, 46].

### Representativeness of sample

Pearson chi-square tests were used to identify bias in participation in the health assessment. Univariate analysis was used to compare participants (*n* = 437 for grip strength; *n* = 468 for TUG) with the total population with medication data available (*n* = 677) on demographics (gender (male/female), age range (44–49 years; 50–64 years; 65+ years), level of ID (mild; moderate; severe and profound), type of residence (independent; community group home; residential care), Down syndrome (yes/no), physical activity level (low; moderate; high) and exposure to medications). This reflected the method used in a previous study of physical fitness measures in adults with ID [69].

### Covariates

*Demographic variables:* Gender (male/female), age range (44–49 years; 50–64 years; 65+ years), level of ID (mild; moderate; severe/profound) and type of residence (independent; community group home; residential care) were included as covariates.

Level of ID is based on reported intelligence quotient (IQ) scores as follows; mild (50–55 to approx. 70), moderate (35–40 to 50–55) and severe/profound (below 35–40) [1]. Participant case notes were used to identify correct classification. Those with unverified level of ID (*n* = 53) were excluded from analysis.

Community group homes were defined as homes with small groups of people with ID (< 10), based in a community setting with staff support. Residential settings were defined where ten or more people share a single living unit or where the living arrangements are campus based. *Physical activity level:* Participants/proxies were asked how many days they had been involved in physical activity in the previous week. The answers were classified into the three categories (low, moderate and high) based on the International Physical Activity Questionnaire (IPAQ) [70]. Low physical activity was defined as no reported activity or some reported activity but not enough to meet moderate or high physical activity criteria as measured by the IPAQ [70, 71].

Moderate activity was defined as either of the following 3 criteria:

- 3 or more days of vigorous activity of at least 20 min per day or
- 5 or more days of moderate-intensity activity and/or walking of at least 30 min per day or
- 5 or more days of any combination of walking, moderate-intensity or vigorous intensity activities achieving a minimum of at least 600 metabolic equivalent (MET) minutes per week.

High activity was defined as either of the following 2 criteria:

- Vigorous-intensity activity on at least 3 days and accumulating at least 1500 MET-minutes per week or
- 7 or more days of any combination of walking, moderate or vigorous intensity activities accumulating at least 3000 MET-minutes/week.

This measure of physical activity captures both work-related and leisure time physical activity.

#### History of falls

Participants/proxies were asked “in the past year have you had any fall including a slip or trip in which you lost your balance and landed on the floor or ground or lower level?” to which they answered “yes”, “no” or “don’t know”. Those who answered “don’t know” were excluded from the analyses (*n* = 10). Answering “yes” to this question was considered a history of falls in the previous 12 months. The measure of history of falls was self-reported.

#### Functional comorbidity index

A modified version of the Functional Comorbidity Index (FCI) was utilised to adjust for comorbidities in the analyses (Additional file 2). The FCI was calculated by summing the presence of a reported doctor’s diagnosis of the following conditions: arthritis; osteoporosis/osteopenia; asthma; lung disease; angina; congestive heart failure (or heart disease); myocardial infarction; neurological disease; stroke or transient ischaemic attack; diabetes mellitus type I or II; upper gastrointestinal disease (e.g. ulcer, hernia, reflux); depression (unipolar or bipolar); anxiety or panic disorder; visual impairment (e.g., cataracts, glaucoma, macular degeneration); hearing impairment; and overweight/obese to produce a continuous score between 0 and 16. Data on osteoporosis/osteopenia and overweight/obese were also supplemented with objective data from the health assessment. Previously, a number of DBI studies have used modified versions of the FCI. Where participants were missing data on two or more conditions (*n* = 145), they were excluded from the FCI score evaluation, reflecting the method used previously [18, 20–22, 28, 45, 46].

### Statistical analysis

Calculation of DBI scores was performed using Microsoft Excel 2010 (Microsoft Corporation). Statistical analyses were performed using Statistical Package for Social Sciences (SPSS) version 21.0 (IBM Corporation). Statistical significance was set at *p* < 0.05.

The characteristics of the study population were described using descriptive analyses (percentages and 95% confidence intervals (CI’s). Medians and interquartile range (IQR) are reported as the data was not normally distributed.

### Rationale for selection of analysis of covariance (ANCOVA)

Techniques for statistical inference can fail in one of two ways: they can incorrectly reject the null hypothesis of no difference between groups (Type I error) or incorrectly fail to reject the null hypothesis of no difference between groups (Type II error) [72]. It has been shown that F-tests (including ANCOVA) are robust to violations of normality in terms of Type I error, considering a wide variety of distributions commonly found in the health and social sciences [73]. Concern over the relative advantages of parametric and non-parametric methods, as a result, has focused on Type II error [72]. The results of comprehensive analysis by Olejnik and Algina (1984) [74] indicated that the parametric analysis of covariance was robust to violations of either the conditional normality or homoscedasticity assumption. In situations where both assumptions were violated, however, and the covariate has a non-normal distribution, the parametric ANCOVA exhibited a slight tendency to lead to a conservative test of the hypothesis when the sample size was small and the nominal level of significance was 0.05 [74]. Much of the literature around use of ANCOVA for non-normal data has focused on baseline and post-exposure data and change scores in randomised studies, as ANCOVA is frequently employed under these circumstances. In these circumstances, ANCOVA has been found to outperform non-parametric methods such as Mann-Whitney for most types of distribution [72]. While Mann-Whitney has been found to outperform ANCOVA in cases of extreme skew in a biomarker study, ANCOVA has still been suggested as the preferred method of analysis for other distributions [72]. ANCOVA also produces a mean score which is more clinically meaningful and interpretable than the medians produced by Mann-Whitney [72]. In addition, semi-parametric and non-parametric alternatives to ANCOVA, such as rank-transform ANCOVA and Quade’s test would not be appropriate for the data in this study as the dependent variable is continuous.

In addition, a key driver behind the choice of ANCOVA for this analysis was its prior use in several studies of the association between physical performance and DBI score [19–21, 45]. This method of analysis has been the accepted procedure for this data previously [19–21, 45], and in order to produce a comparable study it was selected for use in the current analysis, while still giving due consideration to the statistical assumptions of ANCOVA.

### Tests of normality

Observed data is very rarely normally distributed in health science research [75, 76]. This does not invalidate the use of ANCOVA. Tests for normality were performed on the three dependent variables – grip strength (separated by gender), reciprocal TUG and Barthel Index (Table 1).

**Table 1:** Tests of Normality

	Kolmogorov-Smirnov			Shapiro-Wilk		
	Statistic	df	*p* value	Statistic	df	p value
Grip Strength (Female)	0.058	251	0.039	0.987	251	0.024
Grip Strength (Male)	0.065	186	0.055	0.992	186	0.383
Reciprocal TUG (1/TUG)	0.027	468	0.200	0.990	468	0.003
Barthel Index	0.162	635	< 0.001	0.859	635	< 0.001

df: degrees of freedom

**Fig. 2:**
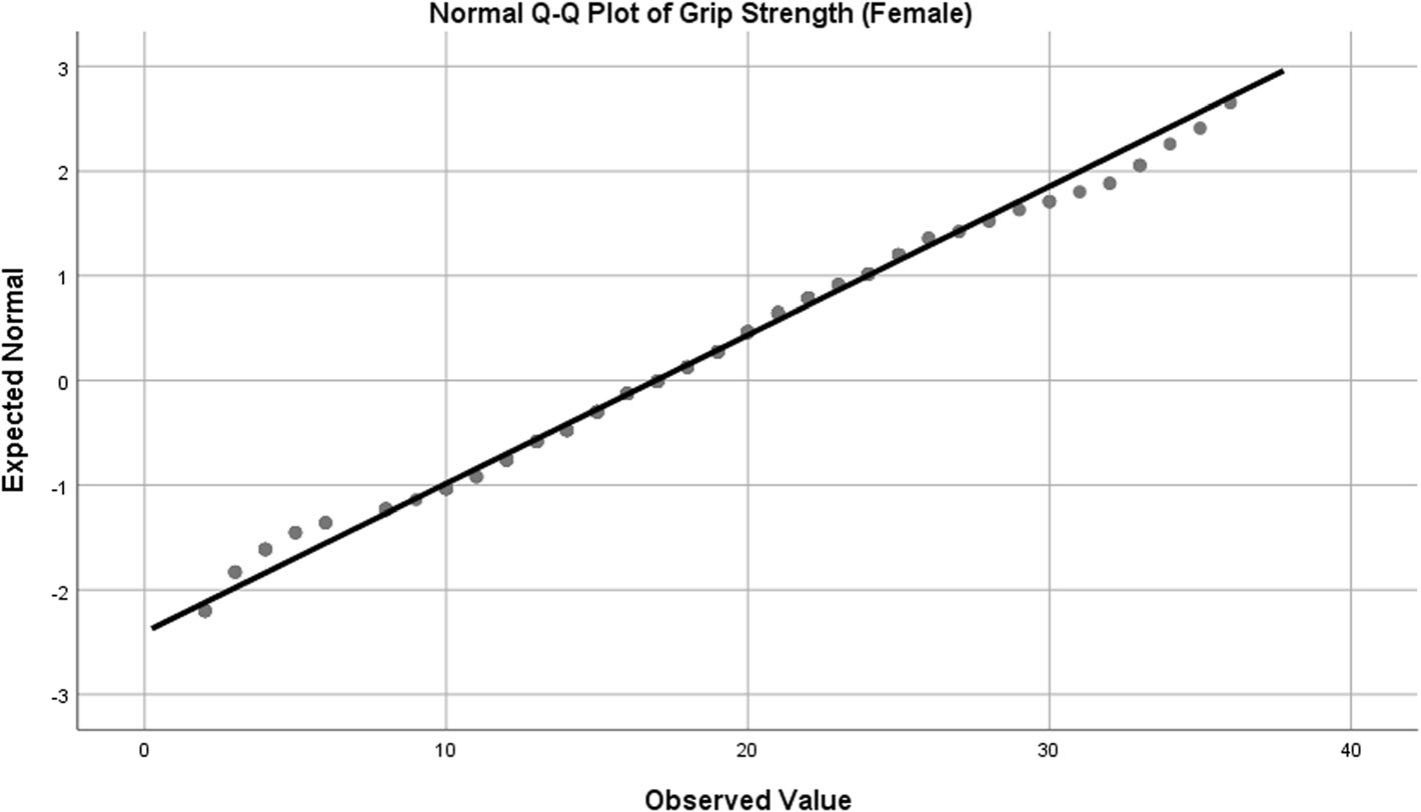
Q-Q Plot for Female Grip Strength

**Fig. 3:**
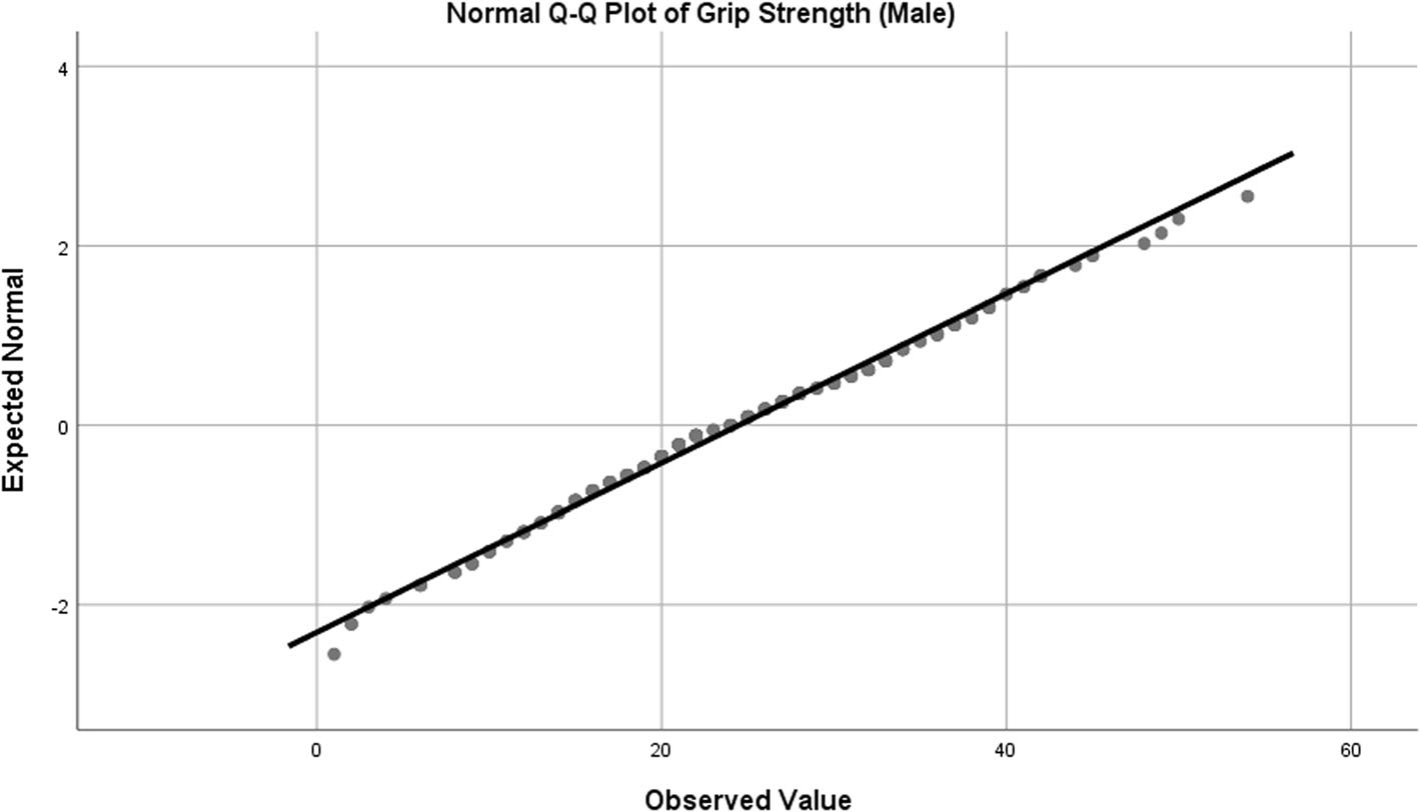
Q-Q Plot for Male Grip Strength

**Fig. 4:**
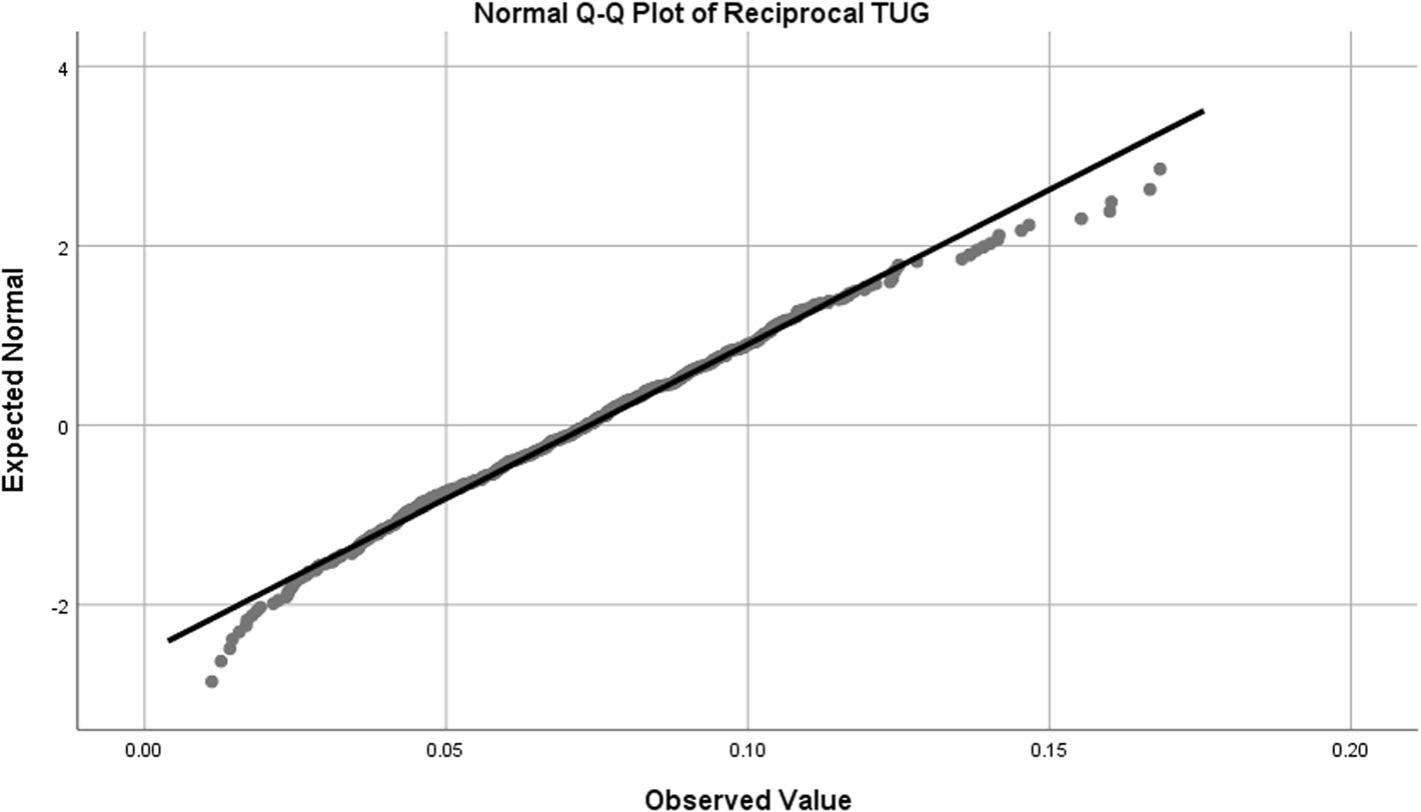
Q-Q Plot for Reciprocal Timed Up and Go

**Fig. 5:**
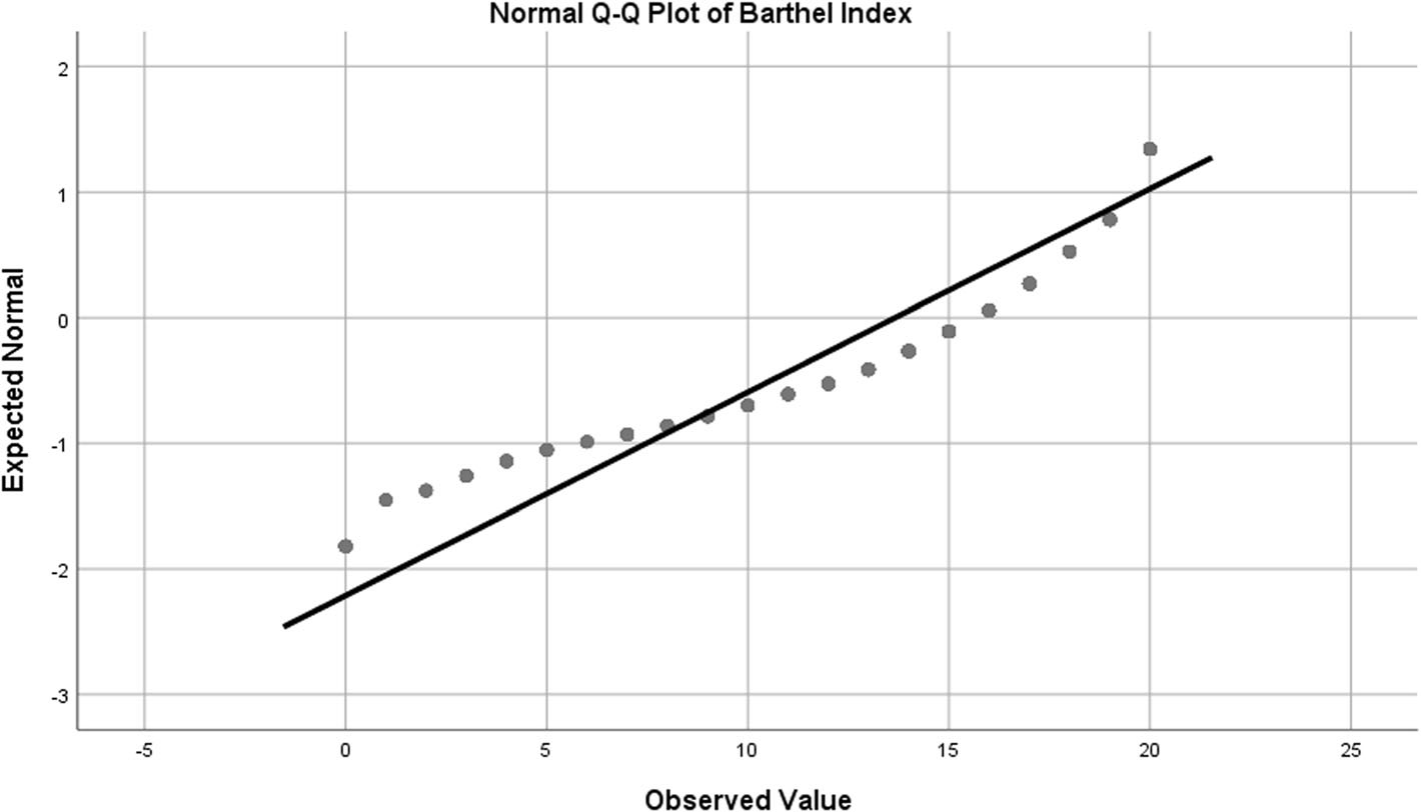
Q-Q Plot for Barthel Index

In addition, normal Q-Q plots were produced in order to visually assess departures from normality. Female grip strength, while statistically significant on statistical normality tests, showed only slight deviation in the Q-Q plot, so were considered to be appropriate for analysis with ANCOVA (Table 1*,* Fig. 2). In the case of male grip strength, both tests used for normality (Kolmogorov-Smirnov and Shapiro-Wilk) were non-significant, indicating a normal distribution (Table 1*,* Fig. 3). For reciprocal TUG, the Kolmogorov-Smirnov test for normality was non-significant, but the Shapiro-Wilk was statistically significant (Table 1*,* Fig. 4). The Kolmogorov-Smirnov test [77] can be used to test for Normality [78, 79]. However, it has been suggested that it may not be as powerful a test for the Normality of data as Anderson-Darling or Shapiro-Wilk [78, 79]. Indeed, Anderson-Darling is a refinement of the Kolmogorov-Smirnov test which gives more weight to the tails, while the Kolmogorov-Smirnov test tends to be more sensitive near the centre than at the tails [78]. Examination of Fig. 4 could explain why the Kolmogorov-Smirnov test of the reciprocal TUG data is not significant, i.e. there was enough evidence to accept the null hypothesis that the distribution is Normal), while the Shapiro-Wilk is significant (suggesting the null hypothesis of Normality should not be accepted) – the data at the tails of the plot appear to be departing from linearity. It is worth noting that the Shapiro-Wilk test can be sensitive to trivial deviations in Normality [80]. However, examination of Fig. 4 displays a very close to Normal distribution. It was concluded, therefore, that for the purposes of this analysis, data for reciprocal TUG could be analysed by ANCOVA. Barthel Index tests for normality were statistically significant, however, after examination of the Q-Q plot for nature of deviation (slight sigmoidal shape), it was considered appropriate to examine ANCOVA (Table 1*,* Fig. 5).

ANCOVA, adjusted for comorbidities, level of ID, Down syndrome and falls, was used to assess the effect of age on mean grip strength for males and females separately. ANCOVA, adjusted for gender, comorbidities, level of ID, and falls, was used to assess the effect of age on TUG.

ANCOVA was also selected to detect associations between drug burden and physical function. This reflects the method of analysis used elsewhere for assessment of association between DBI and physical function measures [19]. Continuous grip strength scores were separated by gender and analysed individually for males and females because of the reported inherent differences in grip strength depending on gender, [81] and because the underlying assumption of Normality for ANCOVA was violated when grip strength was analysed for both males and females together.

TUG scores were reciprocal transformed in order to achieve a Normal distribution and homogeneity of error variances across all groups [82, 83].

ANCOVA was carried out to identify associations between performance in physical function measures and DBI score of > 0, anticholinergic only exposure (DBA > 0), and sedative only exposure (DBS > 0). The reference category was set as those with DBI score = 0. The model was adjusted for demographic variables – age, gender (TUG and Barthel Index), level of ID, history of falls, comorbidities (FCI) and number of non-DBI medicines; grip strength was also adjusted for Down syndrome as a result of the well-documented inferior muscular strength and hypotonia in individuals with Down syndrome [84]. Level of ID and Down syndrome were adjusted for separately as level of ID refers to Intelligence Quotient (IQ) and adaptive behaviour deficits including deficits in intellectual functioning [85] while Down syndrome is an aetiology of ID which encompasses individuals with all levels of ID. TUG was not adjusted for Down syndrome because it measures more than muscle strength alone – it measures balance and executive function and as such was not expected to be as influenced by Down syndrome as grip strength. In addition, the feasibility study carried out by Enkelaar et al. (2013) which examined use of TUG in adults with ID found aetiology of ID was not associated with performance in balance and gait tests [49].

A second ANCOVA was used to compare adjusted means of grip strength, TUG and Barthel Index between participants exposed to three different levels of Drug Burden Index ranges (DBI = 0, DBI 0.1 > 1 and DBI ≥ 1), three levels of anticholinergic exposure (DBA = 0, DBA 0.1 > 1 and DBA ≥ 1) and three levels of sedative exposure (DBS = 0, DBS 0.1 > 1 and DBS ≥ 1). Reciprocal TUG scores were back-transformed after analysis. While use of the anticholinergic and sedative sub-scores of the DBI has not been validated, these have been used previously in order to examine in greater detail associations between DBI and physical function [19]. Giving consideration to the different medication exposure patterns (i.e., higher anticholinergic burden) in older adults with ID, further justified the method of analysis.

Variance Inflation Factors (VIF) and Spearman’s correlation coefficients were used to test independent variables for multicollinearity. All VIFs were < 2, therefore there was no collinearity of concern between variables. Dancy and Reidy’s categorisation [86] was used to interpret Spearman’s correlation coefficients. All correlation coefficient values were < 0.4, indicating only weak correlations existed between variables which were again not of concern.

Power calculations were performed using G*Power (version 3.1.9.2) [87]. To detect a medium effect size (Cohen’s f = 0.25) [88], for three-level analysis (DBI/DBA/DBS 0, 0.1 > 1, ≥1) with 6 covariates, a sample size of 133 for males achieved a power of 0.72 and a sample size of 199 for females achieved a power of 0.89 with α = 0.05 (grip strength assessment). For three-level analysis with 6 covariates, a sample size of 383 achieved a power of 0.995 with α = 0.05 (TUG assessment) and a sample size of 464 achieved a power of 0.999 with α = 0.05 (Barthel Index assessment).

For two-level analysis (DBI/DBA/DBS 0 or > 0) with six covariates, a sample size of 133 for males achieved a power of 0.82 and a sample size of 199 for females achieved a power of 0.94 with α = 0.05 (grip strength assessment). For two-level analysis with 6 covariates, a sample size of 383 achieved a power of 0.998 with α = 0.05 (TUG assessment) and a sample size of 464 achieved a power of 0.999 with α = 0.05 (Barthel Index assessment).

## Results

Table 2 displays descriptive statistics of the characteristics of the IDS-TILDA population and the grip strength and TUG sub-populations.

**Table 2:** Descriptive of characteristics of IDS-TILDA participants (*n* = 677) and of the two physical function measures (grip strength *n* = 437; TUG *n* = 468) and representativeness

Characteristics	IDS-TILDA % (95% CI) (n)	Grip strength % (95% CI) (n)		TUG % (95% CI) (n)	
Total *N*	677	437	*p*	468	*p*
Gender	n = 677	n = 437	*0.355*	n = 468	*0.432*
Male	43.9 (40.2–47.6) (*n* = 297)	42.6 (37.9–47.4) (*n* = 186)		44.9 (40.3–49.5) (*n* = 210)	
Female	56.1 (52.4–59.8) (*n* = 380)	57.4 (52.6–62.1) (*n* = 251)		55.1 (50.5–59.7) (*n* = 258)	
Age range	*n* = 676	*n* = 437	*0.498*	*n* = 468	0.040*
44–49 years	27.6 (24.2–31.0) (*n* = 187)	26.5 (22.5–30.9) (*n* = 116)		28.2 (24.2–32.5) (*n* = 132)	
50–64 years	51.3 (47.5–55.1) (*n* = 347)	51.3 (46.5–56.0) (*n* = 224)		53.4 (48.8–58.0) (*n* = 250)	
65 years +	21.0 (17.9–24.1) (*n* = 142)	22.2 (18.4–26.4) (*n* = 97)		18.4 (15.0–22.2) (*n* = 86)	
Level of ID	*n* = 624	*n* = 398	< 0.001*	*n* = 432	< 0.001*
Mild	23.9 (20.6–27.3) (*n* = 149)	30.2 (25.7–34.9) (*n* = 120)^a^		26.4 (22.3–30.8) (*n* = 114)	
Moderate	44.0 (40.1–47.9) (*n* = 287)	53.3 (48.2–58.3) (*n* = 212)^a^		49.5 (44.7–54.4) (*n* = 214)^a^	
Severe/Profound	30.1 (26.5–33.7) (*n* = 188)	16.6 (13.1–20.6) (*n* = 66)^b^		24.1 (20.1–28.4) (*n* = 104)^b^	
Type of residence	n = 676	n = 437	< 0.001*	n = 468	0.001*
Independent	15.0 (12.3–17.7) (*n* = 102)	17.6 (14.2–21.5) (*n* = 77)		15.8 (12.6–19.4) (*n* = 74)	
Community Group Home	44.1 (40.4–47.8) (*n* = 298)	47.6 (42.8–52.4) (*n* = 208)		47.9 (43.3–52.5) (n = 224)^a^	
Residential Care	40.8 (37.1–44.5) (*n* = 276)	34.8 (30.3–39.5) (*n* = 152)^b^		36.3 (32.0–40.9) (*n* = 170)^b^	
Down syndrome	*n* = 663	n = 437	*0.087*	n = 468	*0.388*
Yes	19.2 (16.2–22.4) (*n* = 127)	16.7 (13.3–20.5) (*n* = 73)		17.3 (14.0–21.0) (*n* = 81)	
No	80.8 (77.6–83.8) (*n* = 536)	81.5 (77.5–85.0) (*n* = 356)		81.0 (77.1–84.4) (*n* = 379)	
Physical activity level	*n* = 668	n = 432	0.043*	*n* = 462	0.002*
Low	73.5 (70.0–76.8) (*n* = 491)	70.6 (66.1–74.9) (*n* = 305)		69.5 (65.1–73.7) (*n* = 321)^b^	
Moderate	24.3 (21.0–27.7) (*n* = 162)	27.3 (23.2–31.8) (*n* = 118)		28.1 (24.1–32.5) (*n* = 130)^a^	
High	2.2 (1.3–3.7) (n = 15)	2.1 (1.0–3.9) (n = 9)		2.4 (1.2–4.2) (n = 11)	
Exposure to any medications	95.1 (93.5–96.7) (*n* = 644)	93.8 (91.1–95.9) (*n* = 410)	*0.249*	94.2 (91.7–96.2) (*n* = 441)	*0.475*
Exposure to DBI medications	78.6 (75.5–81.7) (*n* = 532)	75.3 (71.0–79.3) (*n* = 329)^b^	0.003*	76.7 (72.6–80.5) (*n* = 359)	0.045*
DBI score	n = 677	n = 437	0.017*	n = 468	*0.062*
0	21.4 (18.3–24.5) (*n* = 145)	24.7 (20.7–29.0) (*n* = 108)^a^		23.3 (19.5–27.4) (*n* = 109)	
0.1 > 1	24.4 (21.2–27.6) (*n* = 165)	23.8 (19.9–28.1) (n = 104)		25.4 (21.5–29.6) (*n* = 119)	
≥ 1	54.2 (50.5–58.0) (*n* = 367)	51.5 (46.7–56.3) (*n* = 225)		51.3 (46.7–55.9) (*n* = 240)	
DBA score	n = 677	n = 437	*0.197*	n = 468	*0.685*
0	31.9 (28.4–35.6) (*n* = 216)	33.9 (29.4–38.5) (*n* = 148)		32.5 (28.3–36.9) (n = 152)	
0.1 > 1	33.5 (30.0–37.2) (*n* = 227)	31.4 (27.0–35.9) (*n* = 137)		32.5 (28.3–36.9) (n = 152)	
≥ 1	34.6 (31.0–38.3) (*n* = 234)	34.8 (30.3–39.5) (n = 152)		35.0 (30.7–39.6) (*n* = 164)	
DBS score	n = 677	n = 437	0.039*	n = 468	0.001*
0	50.2 (46.4–54.1) (*n* = 340)	53.8 (49.0–58.5) (*n* = 235)		54.1 (49.4–58.6) (*n* = 253)	
0.1 > 1	30.6 (27.1–34.2) (*n* = 207)	27.9 (23.8–32.4) (*n* = 122)		30.1 (26.0–34.5) (*n* = 141)	
≥ 1	19.2 (16.2–22.2) (n = 130)	18.3 (14.8–22.3) (*n* = 80)		15.8 (12.6–19.4) (n = 74)^b^	

^a^Over-represented ^b^Under-represented

Statistically significant results are marked in bold with an asterisk (*)

Of the 677 individuals who took part in Wave 2 of IDS-TILDA, 56.1% (*n* = 380) were female and 51.3% (*n* = 347) were aged between 50 and 64 years. Of the 624 individuals with data available on level of ID, 44% (*n* = 287) of participants had moderate level of ID; 44% (*n* = 298) of the 676 participants with data on type of residence reported lived in a community group home. Almost three-quarters (73.5%; *n* = 491) of participants reported low level of physical activity. Six hundred forty four participants (95.1%) reported taking medication and 78.6% (*n* = 532) were exposed to medication with anticholinergic and/or sedative activity (DBI medications). 21.4% (*n* = 145) of individuals were not exposed to DBI medicines (DBI = 0), 24.4% (*n* = 165) had a DBI score of 0.1 > 1 and 54.2% (*n* = 367) had a DBI score ≥ 1. On sub-score analysis, 31.9% (*n* = 216) of participants were exposed to no anticholinergic medicines (DBA = 0), 33.5% (*n* = 227) had a DBA score of 0.1 > 1 and 34.6% (*n* = 234) had a DBA score ≥ 1. 50.2% (*n* = 340) of participants were exposed to no sedative medicines (DBS = 0), 30.6% (*n* = 207) had a DBS score of 0.1 > 1 and 19.2% (*n* = 130) had a DBS score ≥ 1 (Table 2).

Table 2 presents univariate analysis of demographic and clinical characteristics and participation in grip strength and TUG measurements. Level of ID, type of residence and physical activity level were significantly associated with participation in both measures (*p* < 0.05). In addition, age range was significantly associated with participation in TUG (*p* = 0.040). Those with mild and moderate ID were over-represented compared to those with severe/profound level of ID in both measures (IDS-TILDA 67.9% for mild/moderate, 30.1% for severe/profound; grip strength 83.5% for mild/moderate, 16.6% for severe/profound, *p* < 0.001; TUG 75.9% for mild/moderate, 24.1% for severe/profound, p < 0.001), and those living in residential care were under-represented in both measures (IDS-TILDA 40.8%; grip strength 34.8%, p < 0.001; TUG 36.3%, *p* = 0.001). Drug Burden Index exposure was significantly different for grip strength participants (*p* = 0.017) but not for TUG participants (*p* = 0.062) when compared to the overall population. DBS scores were statistically significantly different for grip strength and TUG participants when compared to the overall population, and those with high (≥ 1) DBS score were underrepresented. Gender (male/female), Down syndrome (yes/no), medication exposure and DBA scores were not statistically significantly different with regards to participation in either grip strength or TUG (*p* > 0.05).

The mean score for grip strength for women was 16.9 kg (Standard Deviation (SD) ±7.0 kg) and for men was 24.5 kg (SD ±10.6 kg). Mean TUG was 16.8 s (SD ±10.3 s). Mean Barthel Index was 13.7. The range of scores for grip strength was 1 – 54 kg, for TUG was 5.9–89.8 s and for Barthel Index was 0–20. Median and interquartile ranges (IQR) are also reported (grip strength for women median score 17 kg, IQR 9 kg; grip strength for men 24 kg, IQR 16.25 kg; TUG median score 13.6 s, IQR 7.1 s; Barthel Index median score 16.0, IQR 9.0) (Table 3*).*

**Table 3:** Data for Grip Strength, TUG and Barthel Index

Health Measure	Mean	±SD	Range (min – max)	Median	Interquartile Range
Grip strength (females) (kg) (n = 251)	16.9	7.0	2.0–36.0	17.0	9.0
Grip strength (males) (kg) (n = 186)	24.5	10.6	1.0–54.0	24.0	16.25
TUG (seconds) (n = 468)	16.8	10.3	5.9–89.8	13.6	7.1
Barthel Index (*n* = 635)	13.7	6.2	0.0–20.0	16.0	9.0

Grip strength data was categorised by age and gender according to the manufacturer’s instructions (Table 4*).* Four hundred eight participants (93.4%) were categorised as having “Below Normal” grip strength, 15 (3.4%) had “Normal” grip strength and 14 (3.2%) had “Above Normal” grip strength.

**Table 4:** Categorical Data for Grip Strength (n = 437)

Grip Strength (n = 437)	% of Participants (95% CI)
Above Normal (n = 14)	3.2 (1.8–5.3)
Normal (n = 15)	3.4 (1.9–5.6)
Below Normal (*n* = 408)	93.4 (90.6–95.5)

**Fig. 6:**
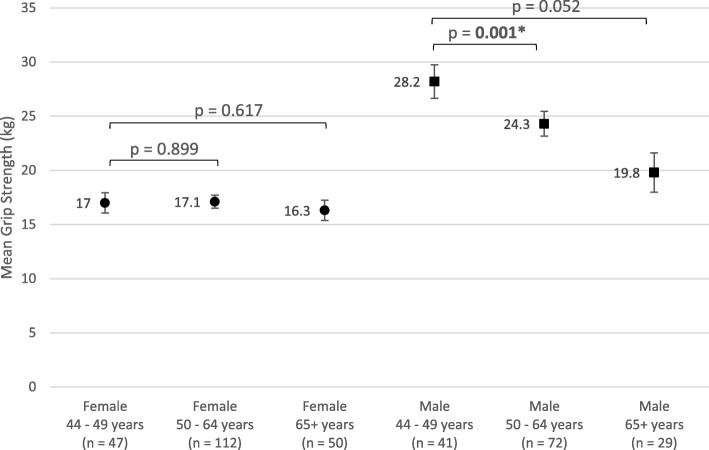
Grip Strength Versus Age and Gender

For women, there is no significant association of age with grip strength after adjusting for confounders (comorbidities, level of ID, Down syndrome and falls), *p* > 0.05, Fig. 6.

For men, there is a highly significant association of age with grip strength, with mean grip strength decreasing from 28.2 kg at 44–49 years to 24.3 kg at 50–64 years (*p* = 0.001) to 19.8 kg at 65+ years (*p* = 0.052) (Fig. 6*)*.

**Fig. 7:**
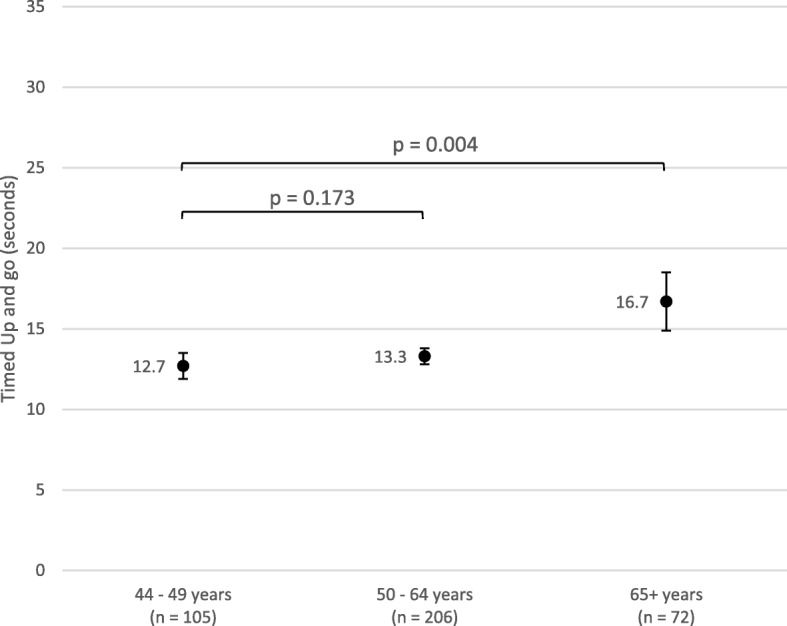
Timed Up and Go Versus Age

There is no significant association of age with increased TUG score from age range 44–49 years to 50–64 years after adjusting for confounders (gender, comorbidities, level of ID and falls), *p* > 0.05, Fig. 7. However, there is a statistically significant association of age range with increased TUG between the youngest and oldest age ranges (44–49 years and 65+ years), with mean TUG increasing from 12.7 s at 44–49 years to 16.7 s at 65+ years (*p* = 0.004) (Fig. 7*)*.

Table 5 displays results from unadjusted and adjusted ANCOVA models for grip strength, TUG and Barthel Index in those exposed to DBI, DBA and DBS medications (> 0) versus those not exposed (= 0). Adjusted grip strength scores for females or males were not significantly associated with DBI, DBA or DBS exposure (*p* > 0.05 in all cases). Unadjusted reciprocal TUG scores were significantly associated with DBI, DBA and DBS scores > 0 (DBI > 0, *p* = 0.010; DBA > 0, *p* = 0.016; DBS > 0, p = 0.001). However, after adjusting for confounding factors, this association was no longer observed (p > 0.05 in all three analyses). Unadjusted Barthel Index was significantly associated with DBI, DBA and DBS scores > 0 (*p* < 0.001 in all three cases). After adjusting for confounders, Barthel Index remained significantly associated with DBS > 0 (*p* = 0.005).

**Table 5:** Analysis of Covariance between DBI, DBA, DBS and Grip Strength, Reciprocal TUG and BI

	DBI > 0			DBA > 0			DBS > 0		
	B (95% CI)	p	n	B (95% CI)	p	n	B (95% CI)	p	n
Grip strength (females)									
n = 251	−0.937 (−3.083; 1.209)	0.391	198	−0.252 (−2.122; 1.618)	0.791	169	−2.048 (−3.784; − 0.313)	0.021*	125
*n* = 209*	0.998 (−1.240; 3.235)	0.380	162	1.224 (−0.720; 3.168)	0.216	139	−0.557 (−2.418; 1.303)	0.555	108
Grip strength (males)									
n = 186	−2.062 (−5.418; 1.295)	0.227	131	−1.358 (−4.566; 1.850)	0.405	120	1.126 (−1.992; 4.244)	0.477	77
n = 142*	0.465 (−3.470; 4.400)	0.816	102	1.430 (−2.251; 5.111)	0.444	94	3.064 (−0.512; 6.641)	0.092	60
Reciprocal TUG									
n = 468	−0.008 (− 0.014; − 0.002)	0.010*	359	−0.007 (− 0.013; − 0.001)	0.016*	316	−0.009 (− 0.014; − 0.004)	0.001*	215
*n* = 383*	0.002 (− 0.004; 0.009)	0.517	295	0.002 (− 0.004; 0.008)	0.512	261	− 0.003 (− 0.009; 0.002)	0.241	184
Barthel Index									
n = 635	−3.561 (−4.705; −2.417)	< 0.001*	500	−2.467 (− 3.487; −1.447)	< 0.001*	436	− 3.275 (− 4.204; − 2.346)	< 0.001*	315
*n* = 464*	− 1.060 (− 2.285; 0.165)	0.090	369	−0.782 (− 1.837; 0.273)	0.146	323	−1.438 (− 2.435; − 0.440)	0.005*	238

*For each end-point the first row represents unadjusted model and second row adjusted model for age, level of ID, history of falls, Functional Comorbidity Index, number of non-DBI medicines. Grip strength is also adjusted for Down syndrome. TUG and Barthel Index are adjusted for gender. B, parameter estimate; CI, confidence interval; DBI, Drug Burden Index; DBA, anticholinergic component of Drug Burden Index; DBS, sedative component of Drug Burden Index; p, significance; n, number exposed to anticholinergic and/or sedative drugs. n first row, number of subjects completing each test; n second row represents available data on subjects after controlling for confounders

**Fig. 8:**
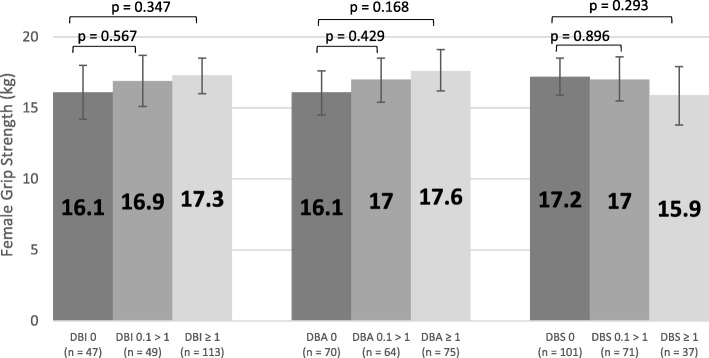
Adjusted Mean Scores for Female Grip Strength

**Fig. 9:**
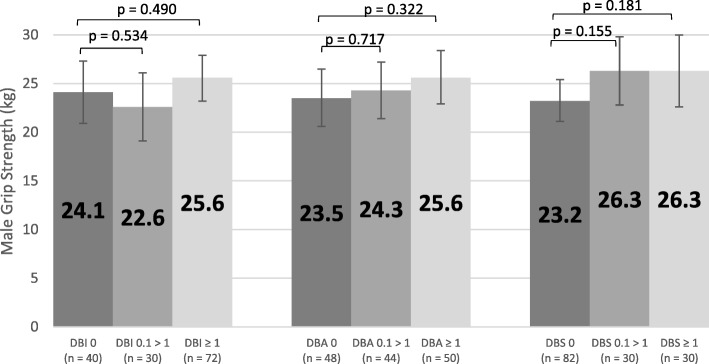
Adjusted Mean Scores for Male Grip Strength

**Fig. 10:**
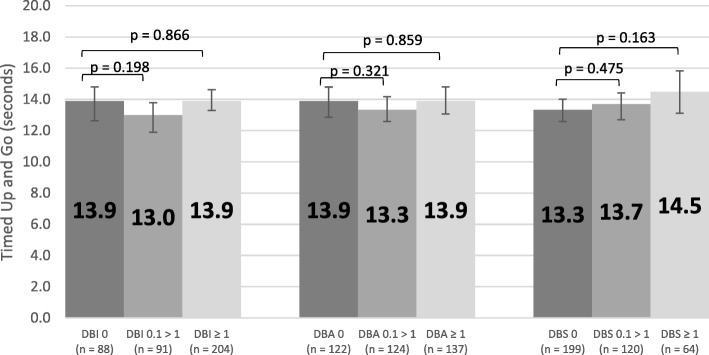
Adjusted Mean Scores for Timed Up and Go

Figure 8 displays the adjusted mean scores for female grip strength across the three types of exposure (= 0, 0.1 > 1, ≥ 1) for DBI, DBA and DBS. There was no significant association between exposure levels and grip strength scores (p > 0.05). Similarly, Fig. 9 displays adjusted mean scores for male grip strength, which were also not significantly associated with exposure level (p > 0.05). Adjusted reciprocal TUG scores were back transformed after analysis and there was no significant association between exposure levels and scores (p > 0.05, Fig. 10).

**Fig. 11:**
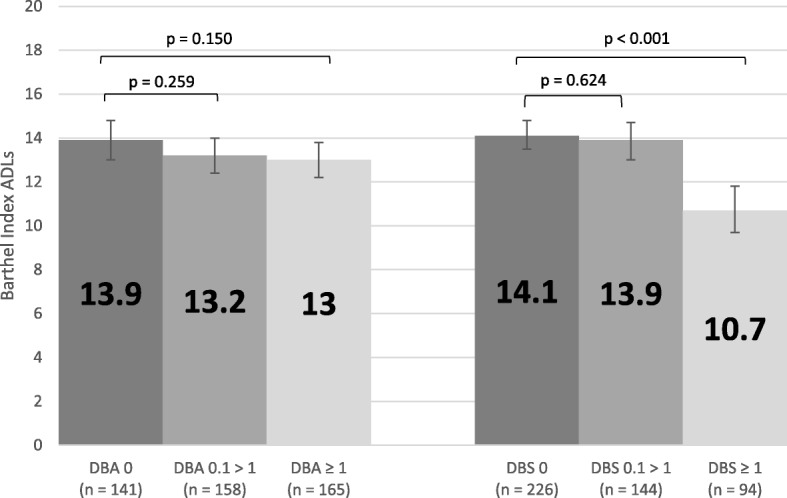
Adjusted Mean Scores for Barthel Index

Increased dependency in Barthel Index activities of daily living was not significantly associated with DBA exposure after adjusting for confounders (p > 0.05), but increased dependency was significantly associated with DBS exposure (p < 0.001 for DBS = 0 vs DBS ≥ 1, Fig. 11).

## Discussion

### Key findings

Drug Burden Index was not associated with poorer performance in two measures of physical function, grip strength and TUG, in analysis of covariance after adjusting for confounding factors in this cross-sectional study in older adults with ID. On sub-score analysis, the anticholinergic only and sedative only components of the DBI were also not associated with poorer performance in these measures.

### Grip strength

Grip strength scores were lower in the IDS-TILDA population than in the Dutch cross-sectional study “Healthy ageing and intellectual disabilities” (HA-ID), which included participants aged 50 years and over living primarily in residential care in the Netherlands (*n* = 1050, of which 725 completed grip strength assessment) [69, 89]. In particular, grip strength scores for women in all age groups were lower than their counterparts in HA-ID *(*Additional file 3*)* [69]. Variation of grip strength scores was also large, with scores ranging from 2.0 kg to 36.0 kg (Table 3). It is important to consider that 9 out of 10 participants were categorised as having “Below Normal” grip strength. This finding is not surprising - Hilgenkamp et al. [69] compared reference values for grip strength by gender in the general population with scores from older adults with intellectual disabilities living primarily in residential care settings. They found that 76.5% of men and 67.3% of women with ID had below average grip strength scores, compared to 2.5% of older adults in the general population.

It has been suggested that grip strength may be too low at baseline in this population to be able to find predictive relationships with measures including daily functioning [69, 90].

Female grip strength was significantly associated with exposure to DBS medications in the unadjusted model (*p* = 0.021, Table 5). However, after adjusting for confounding factors, this association was no longer statistically significant (*p* > 0.05). DBI, DBA and DBS were not significantly associated with grip strength in the other unadjusted or adjusted models (Table 5).

### Timed up and go

TUG scores in IDS-TILDA were comparable to other studies of older adults with ID. The mean TUG score in a Dutch observational feasibility study of 76 adults aged 50 years and over with mild to moderate ID was also 17 s, although this study excluded individuals with severe/profound ID and those with epilepsy from the analysis [49]. There was large variation in scores across the population in IDS-TILDA for TUG, with a range of values between 5.9 and 89.8 s.

Our study findings revealed that while there was a significant association between timed up and go scores and exposure to DBI, DBA and DBS medications in the unadjusted models (*p* = 0.010, 0.016 and 0.001, respectively, Table 5), after adjusting for confounding factors this association was no longer statistically significant (p > 0.05).

### Barthel index activities of daily living

Previously, DBI exposure was found to be significantly associated with a decline in Barthel Index score in this cohort [15]. The current study provides a more in-depth analysis of the two components of DBI, and found that after adjusting for confounding factors, sedative drug burden was significantly associated with increased dependence in Barthel Index activities of daily living (*p* < 0.001, Fig. 11), but anticholinergic drug burden was not significantly associated with Barthel Index scores (p > 0.05, Fig. 11).

### Comparison with other studies

Grip strength scores were lower than findings from The Irish Longitudinal Study on Ageing (TILDA), which follows community-dwelling older adults without ID aged 50 years and over (*n* = 5897, of which 5819 completed grip strength assessment) [69, 89, 91] (Additional files 3 and 4).

Similarly, TUG scores in IDS-TILDA were also poorer than those observed in older adults without ID. TILDA reported mean TUG scores of 10 s [92], while in IDS-TILDA, the unadjusted mean score was 17 s (Table 3 and Additional file 5). There was greater variation in scores (reflected by standard deviation) for both physical function measures in IDS-TILDA when compared to TILDA, and this type of variation is similar to that seen in the HA-ID population [69, 91].

An association between higher DBI scores and lower grip strength has been reported in older adults without ID elsewhere [19, 20]. The existing evidence in the literature suggests there is an association between higher DBI scores and slower TUG in older adults without ID [45].

A number of factors could be influencing the physical function scores of older adults with ID; ageing, gender, physical activity level, medication burden and the presence of the intellectual disability itself.

It is important to note that the age range encompassed by this study (age ≥ 44 years) represents a younger cohort than that typically investigated in studies of ageing. However, because people with ID experience the onset of age-related conditions at a much younger age than the general population, and also experience premature ageing and reduced life expectancy (in Ireland, life expectancy at birth has been found to be 19 years lower than for people without ID, and death occurs earlier the more severe the level of ID [93, 94]), choice of this younger cohort reflects the relatively new phenomenon of adults with ID living into older age [2, 6]. The effect of ageing, which is more pronounced at a younger age in people with ID, combined with the long term reduced activity levels, could be overpowering the effect of drug burden alone. Decline in grip strength is significantly associated with age for men in this cohort, with mean grip strength decreasing across the three age ranges after adjusting for confounding factors (*p* ≤ 0.05); however, there is no significant association between decline in grip strength and age range for women (*p* > 0.05, Fig. 6).

This also suggests that gender is an important factor when examining decline in grip strength, as men appear to be more susceptible to decline as they age, while women, though producing lower scores overall, maintain their function in this area.

Almost three quarters of the IDS-TILDA cohort have reported low levels of physical activity *(*Table 2*),* and this may be contributing to the reduced muscle strength and balance observed in this study. The low physical fitness level may be a result of lifelong sedentary lifestyle [69]. It is well-established that adults with ID are less physically active than their counterparts without ID, and this lower level of activity is evident across all age ranges [95–97]. It has been suggested that a lack of adequate physical activity choices in day services and residential care settings prevents adults with ID from meeting recommendations for physical activity [98]. However, improvements in physical activity, such as cardiovascular training and balance and weight-bearing exercises lead to better performance in tests of muscle strength and improvement in measures of balance and executive function, including TUG [95]. This suggests that physical activity levels play a substantial role in the extent of physical function, and that long term inactivity impacts greatly on performance in tests of function.

Adults with ID generally take medications with anticholinergic and sedative effects throughout their lifetime, while older adults in the general population may only begin to take these classes of medications later in life [99]. People with ID are exposed to higher proportions of these medications for a longer duration of time. Therefore, the effect on performance for older adults without ID is more pronounced from these types of medications. In previous DBI studies of older adults without ID, sedative medications contributed more to the overall Drug Burden Index score than anticholinergic medications, while in recently published research in the IDS-TILDA cohort, scores were considerably higher and anticholinergic medications contributed more to the burden [15]. The association with physical function has been found to be stronger for the sedative sub-score of the DBI than the anticholinergic sub-score in older adults without ID [19]. Interestingly, neither number of medications nor use of psychotropic medication were associated with any of the balance and gait tests carried out in a study of older adults with mild to moderate ID in the Netherlands [49]. Older adults with ID also differ from those without ID because they have higher levels of polypharmacy even in the youngest age group [13, 100] and the evidence in the literature suggests that this higher level of drug use begins much earlier [100, 101]. If exposure to DBI medications makes a contribution, it may be much earlier in the life of people with ID.

It may also be that the onset of epilepsy, dementia and neurological impairment, which is more prevalent in adults with ID as they age [7, 11, 69], causes a rapid decline in physical functioning, and the response of carers to these changes and their influence on the possibilities for physical activity in the person with ID, would render drug effects small in comparison.

The association between sedative drug burden and increased dependency in Barthel Index activities of daily living is of particular interest because the association remained even after correcting for the factors which may be having a strong influence on physical function, such as age and gender. It has been suggested that association of total DBI, anticholinergic-only Drug Burden and sedative-only Drug Burden with poorer Barthel Index is independent of established determinants of poorer physical function and the magnitude of the associations were similar to that of these determinants [102]. Targeting sedative medications for review and re-assessing Barthel Index scores could potentially identify an area for clinical intervention that may improve quality of life in older adults with ID.

### Strengths and limitations

There are four main strengths in this study. First, this study uses data from a large, nationally representative sample of older adults with ID. Second, comprehensive medication data was collected for this cohort. Third, the Drug Burden Index was used, which is an internationally validated, robust measure of anticholinergic and sedative drug effects. Fourth, objective measures of physical performance were measured.

However, there are also several limitations to this study. This is a cross-sectional observational study so it is only possible to describe association, not causality. Another possible limitation of this study is that it under-represents those with severe/profound level of ID. It has been suggested that the higher drop-off rate from participants with severe/profound level of ID in grip strength measurements is as a result of difficulty in adaptation to grasping for this group [103]. There is also no baseline/pre-exposure data, as these adults have typically been taking medications with anticholinergic and/or sedative effects long-term. The use of sub-score analysis of anticholinergic-only burden and sedative-only burden scores has not been validated in relation to physical function measures in any population. Finally, some of the data obtained is based on reported rather than measured data, and this may be a limitation to the accuracy of some data.

## Conclusions

The current study findings suggest that the DBI tool may not be useful at identifying the effect of these medications on physical function in older adults with ID. However, the adverse effects of these medications are well established. It is highly probable that these medications are indeed affecting function to a degree in this cohort, but that this effect occurs earlier in life in these individuals. As this study includes individuals aged 44 years and older, there is no reference available from the current study design to examine function among younger adults with ID. It is possible that exposure to anticholinergic and sedative medications at a younger age leads to lifelong exposure, which then impacts on physical function. The lack of pre-exposure data from the study design limits the understanding of whether exposure to these medications have an immediate or permanent effect on physical performance. This is an area with potential for further research in future. It is likely that the DBI tool is useful for detecting changes in physical function in older adults without ID that commence anticholinergic and/or sedative medications later in life. Regular medication review should be carried out for older adults with ID, alongside appropriate de-prescribing, due to the well-established adverse effects and interaction potential for these types of medications. The higher morbidity in this population also suggests that they are more likely to be exposed to these medications, and while the reference category of those with DBI = 0 have, at this point in time, no exposure to these medications, it does not necessarily mean they were never exposed, and it is not possible to ascertain prior exposure and duration of treatment from the current study design.

Considering the substantial number of participants who did not wish to, or were not able to complete the tests, this cohort of older adults with ID appear less active and physically weaker than older adults without ID. This also poses challenges to determining if there is a further decline in their physical functioning associated with drug use because of the low level of physical function they have attained already.

While DBI may be associated with other outcomes in older adults with ID, including higher dependence in Barthel Index activities of daily living [15], it was not significantly associated with the two objective measures of physical function in this study. Further study is needed to investigate associations between DBI and physical function in adults with ID, potentially by following a younger cohort, aged 40–50 years, over a longer period of time.

## Additional files

Additional file 1: Modified Barthel Index. A descriptive table of the Barthel Index components matched to variables on function from The Intellectual Disability Supplement to the Irish Longitudinal Study on Ageing (IDS-TILDA). (DOCX 50 kb)

Additional file 2: Modified Functional Comorbidity Index. A descriptive table of the Functional Comorbidity Index components matched to variables on morbidities from The Intellectual Disability Supplement to the Irish Longitudinal Study on Ageing (IDS-TILDA). (DOCX 15 kb)

Additional file 3: Female Grip Strength Comparison. A comparative table of female grip strength scores from The Intellectual Disability Supplement to the Irish Longitudinal Study on Ageing (IDS-TILDA), the Healthy ageing and intellectual disabilities study (HA-ID) and the Irish Longitudinal Study on Ageing (TILDA). (DOCX 14 kb)

Additional file 4: Male Grip Strength Comparison. A comparative table of male grip strength scores from The Intellectual Disability Supplement to the Irish Longitudinal Study on Ageing (IDS-TILDA), the Healthy ageing and intellectual disabilities study (HA-ID) and the Irish Longitudinal Study on Ageing (TILDA). (DOCX 16 kb)

Additional file 5: Timed Up and Go Comparison. A comparative table of timed up and go scores from The Intellectual Disability Supplement to the Irish Longitudinal Study on Ageing (IDS-TILDA) and the Irish Longitudinal Study on Ageing (TILDA). (DOCX 14 kb)

## Data Availability

The datasets generated and/or analysed during the current study are not publicly available. The data used for this study contains sensitive information on the cohort of older adults with ID. Currently, the IDS-TILDA dataset is only available with the permission of the Principal Investigators. Due to the sensitive nature of the data, strict data protection protocols are in place to manage and control the access to this data. Access to this data is only available through a hot desk system under the permission and discretion of the Principal Investigators.

## Abbreviations

ANCOVA: Analysis of Covariance
ATC: Anatomical Therapeutic Chemical Classification System
BI: Barthel Index
BMI: Body Mass Index
CAPI: Computer Assisted Personal Interview
DBA: Anticholinergic Subscale of Drug Burden Index
DBI: Drug Burden Index
DBS: Sedative Subscale of Drug Burden Index
df: degrees of freedom
FCI: Functional Comorbidity Index
HA-ID: Healthy Ageing and Intellectual Disabilities
HPRA: Health Products Regulatory Authority
ID: Intellectual Disabilities
IDS-TILDA: Intellectual Disability Supplement to the Irish Longitudinal Study on Ageing
INN: International Non-Proprietary Name
IPAQ: International Physical Activity Questionnaire
IQ: Intelligence Quotient
IQR: Interquartile Range
MDD: Minimum Daily Dose
NIDD: National Intellectual Disability Database
OTC: Over the Counter
PIN: Personal Identification Numbers
PIQ: Pre-Interview Questionnaire
QUS: Quantitative Ultrasound
RNID: Registered Nurse in Intellectual Disability
SD: Standard Deviation
SmPC: Summary of Product Characteristics
SPSS: Statistical Package for Social Sciences
STROBE: Strengthening the Reporting of Observational Studies in Epidemiology
TUG: Timed up and go
VIF: Variance Inflation Factors
