# Supplementary material for: Association of Drug Burden Index with grip strength, timed up and go and Barthel index activities of daily living in older adults with intellectual disabilities: an observational cross-sectional study
Source: BMC Geriatr. 2019 Jun 24;19:173. doi: 10.1186/s12877-019-1190-3 (PMC6591943; doi:10.1186/s12877-019-1190-3)
Supplement: Supplementary file 1 — Modified Barthel Index. A descriptive table of the Barthel Index components matched to variables on function from The Intellectual Disability Supplement to the Irish Longitudinal Study on Ageing (IDS-TILDA). (DOCX 50 kb) [file 12877_2019_1190_MOESM1_ESM.docx]

| **IDS-TILDA Question** | **IDS-TILDA Codes** | **Modified to** | **Modified Barthel Index Codes** |
| --- | --- | --- | --- |
| **Mobility** |  |  |  |
| Please indicate the level of difficulty, if any, you have with walking 100 yards | 1=No difficulty |  | 3=Independent |
|  | 2=Some difficulty |  | 2=Walks with help |
|  | 3=A lot of difficulty |  | 1=Wheelchair independent |
|  | 4=Cannot do at all |  | 0=Immobile |
| **Stairs** | | |  |
| Please indicate the level of difficulty, if any, you have with climbing one flight of stairs without resting. | 1=No difficulty |  | 2=Independent |
|  | 2=Some difficulty |  | 1=Needs help |
|  | 3=A lot of difficulty |  | 0=Unable |
|  | 4=Cannot do at all |  |  |
| **Dressing** |  |  |  |
| Please indicate the level of difficulty, if any, you have with dressing, including putting on shoe and socks. | 1=No difficulty |  | 2= Independent |
|  | 2=Some difficulty |  | 1= Needs help |
|  | 3=A lot of difficulty |  | 0=Unable |
|  | 4=Cannot do at all |  |  |
| **Bathing** |  |  |  |
| Please indicate the level of difficulty, if any, you have with bathing or showering. | 1=No difficulty |  | 1= Independent |
|  | 2=Some difficulty |  | 0= Dependent |
|  | 3=A lot of difficulty |  |  |
|  | 4=Cannot do at all |  |  |
| **Grooming** |  |  |  |
| Please indicate the level of difficulty, if any, you have with cleaning your teeth/taking care of your dentures. | 1=No difficulty |  | 1= Independent |
|  | 2=Some difficulty |  | 0= Needs help |
|  | 3=A lot of difficulty |  |  |
|  | 4=Cannot do at all |  |  |
| **Feeding** |  |  |  |
| Please indicate the level of difficulty, if any, you have with eating such as cutting up your food, use of utensils, drinking from a cup/glass etc? | 1=No difficulty |  | 2=Independent |
|  | 2=Some difficulty |  | 1=Needs help |
|  | 3=A lot of difficulty |  | 0= Dependent |
|  | 4=Cannot do at all |  |  |
| **Transfer** |  |  |  |
| Please indicate the level of difficulty, if any, you have with getting in or out of bed. | 1=No difficulty |  | 3= Independent |
|  | 2=Some difficulty |  | 2=Minor Help |
|  | 3=A lot of difficulty |  | 1 =Major help |
|  | 4=Cannot do at all |  | 0=Unable |
| **Toileting** |  |  |  |
| Please indicate the level of difficulty, if any, you have with using the toilet, including getting up or down. | 1=No difficulty |  | 2=Independent |
|  | 2=Some difficulty |  | 1=Needs help |
|  | 3=A lot of difficulty |  | 0= Dependent |
|  | 4=Cannot do at all |  |  |
| **Bladder Continence** |  |  |  |
| During the last 12 months, have you lost any amount of urine beyond your control? | Yes |  | 0=Incontinent |
| Did this happen more than once during a 1 month period? | No |  | 1=Occasional (In last month) |
| During the last 12 months, have you lost any amount of urine beyond your control? | No |  | Continent |
| **Bowel Continence** |  |  |  |
| During the last 12 months, have you lost any amount of faeces beyond your control? | Yes |  | 0=Incontinent |
| During the last 12 months, have you lost any amount of urine beyond your control? | No |  | 1=Occasional (In last month) |
| During the last 12 months, have you lost any amount of faeces beyond your control? | No |  | Continent |

Additional file 1: Modified Barthel Index
