# Supplementary material for: Association of Drug Burden Index with grip strength, timed up and go and Barthel index activities of daily living in older adults with intellectual disabilities: an observational cross-sectional study
Source: BMC Geriatr. 2019 Jun 24;19:173. doi: 10.1186/s12877-019-1190-3 (PMC6591943; doi:10.1186/s12877-019-1190-3)
Supplement: Supplementary file 2 — Modified Functional Comorbidity Index. A descriptive table of the Functional Comorbidity Index components matched to variables on morbidities from The Intellectual Disability Supplement to the Irish Longitudinal Study on Ageing (IDS-TILDA). (DOCX 15 kb) [file 12877_2019_1190_MOESM2_ESM.docx]

| **FCI Item** | **IDS-TILDA Variable** | | **Score** |
| --- | --- | --- | --- |
| Arthritis (rheumatoid arthritis and osteoarthritis) | *Has arthritis (including osteoarthritis or rheumatism)* | | 1 |
| Osteoporosis | *Has Osteoporosis/thin or brittle bones*  *Has QUS evidence for Osteoporosis and Osteopenia*  *Has scoliosis (all participants with scoliosis had evidence of osteoporosis/osteopenia)* | | 1 |
| Asthma | *Has Asthma* | | 1 |
| COPD, ARDS, emphysema | *Has chronic lung disease* | | 1 |
| Angina | *Has had angina* | | 1 |
| Congestive heart failure (or heart disease) | *Has congestive heart failure  Has a heart murmur Has an abnormal heart rhythm  Have had angioplasty or stent Have ever had open heart surgery Has other heart trouble* | | 1 |
| Heart attack (MI) | *Has had a heart attack* | | 1 |
| Neurological disease (e.g. MS, Parkinson's) | *Has Parkinson's disease/  Has Multiple sclerosis/  Has Alzheimer's disease/  Has Spina bifida/ Has dementia, organic brain syndrome or senility/  Has epilepsy/ Has Cerebral Palsy/ Has muscular dystrophy* | | 1 |
| Stroke or TIA | *Has had stroke  Has had ministroke/TIA* | | 1 |
| Peripheral vascular disease/ peripheral artery disease |  | | **Excluded^a^** |
| Diabetes I or II | *Has diabetes*  *Diabetic diet* | | 1 |
| Upper GI disease (e.g. ulcer, hernia, reflux) | *Has Gastroesophageal reflux disease/ Has stomach ulcers* | | 1 |
| Depression | *Has depression*  *Has manic depression* | | 1 |
| Anxiety or panic disorder | *Has anxiety condition* | | 1 |
| Visual impairment (e.g., cataracts, glaucoma, macular degeneration) | *Has age related macular degeneration/  Has glaucoma/  Has cataracts/ Has had cataracts surgery Has other eye disease/ Have you been prescribed glasses or contact lenses?* | | 1 |
| Hearing impairment (i.e. very hard of hearing, even with hearing aids) | *Is your hearing (with or without a hearing aid)...* ***fair/poor?***  *Use of: Hearing aid (all of the time)*  *Hearing aid (some of the time)*  *Phone messaging service*  *Amplifier* | | 1 |
| Degenerative disc disease (e.g. back disease, spinal stenosis, or severe chronic back pain) |  | | **Excluded^a^** |
| Overweight/Obese^b^ | *Aggregated score of objective measure of:*   - *BMI (calculated from height and weight)* - *Weight and ulnar length to estimate height (where height could not be measured)* - *Mid Upper Arm Circumference measure (where could not measure weight or height eg wheelchair user)*   *Weight reducing diet* | | 1 |
|  |  | **MAX SCORE=16** | |
| 1. Excluded as there was inadequate participant data collected to capture this condition. 2. These two categories were aggregated as the Mid Upper Arm Circumference measure yields only three categories of result: underweight, normal and overweight/obese. | | | |

Additional file 2: Modified Functional Comorbidity Index
