# Supplementary material for: Association of Drug Burden Index with grip strength, timed up and go and Barthel index activities of daily living in older adults with intellectual disabilities: an observational cross-sectional study
Source: BMC Geriatr. 2019 Jun 24;19:173. doi: 10.1186/s12877-019-1190-3 (PMC6591943; doi:10.1186/s12877-019-1190-3)
Supplement: Supplementary file 3 — Female Grip Strength Comparison. A comparative table of female grip strength scores from The Intellectual Disability Supplement to the Irish Longitudinal Study on Ageing (IDS-TILDA), the Healthy ageing and intellectual disabilities study (HA-ID) and the Irish Longitudinal Study on Ageing (TILDA). (DOCX 14 kb) [file 12877_2019_1190_MOESM3_ESM.docx]

Additional file 3: Female Grip Strength Comparison

| **Age Range** | **IDS-TILDA Female (n = 251)** | | **HA-ID Female**  **(n = 355)** | | **TILDA Female < 160cm** | | **TILDA Female ≥ 160cm** | |
| --- | --- | --- | --- | --- | --- | --- | --- | --- |
|  | *n* | *Mean Grip Strength (kg)* | *n* | *Mean Grip Strength (kg)* | *Age* | *Mean Grip Strength (kg)* | *Age* | *Mean Grip Strength (kg)* |
| 44 – 49  50 – 54  55 – 59  60 – 64  65 – 69  70 – 74  75 – 79  80 – 84  85 – 89 | 60  53  48  29  35  14  8  4  0 | 16.60 ± 8.3  19.49 ± 5.8  17.67 ± 6.7  14.27 ± 6.64  16.23 ± 5.7  16.64 ± 8.4  14.63 ± 4.8  10.75 ± 8.0  N/A | 0  82  90  65  47  38  23  5  5 | N/A  21.34 ± 8.83  20.23 ± 7.97  20.08 ±7.13  21.34 ± 5.58  17.92 ± 6.47  18.91 ± 7.33  17.4 ± 6.11  14.8 ± 5.72 | N/A  50  55  60  65  70  75  80  85 | N/A  23.0 ± 4.6  21.3 ± 4.7  20.7 ± 4.5  20.3 ± 4.2  19.1 ± 3.9  17.9 ± 3.9  16.8 ± 4.1  15.7 ± 4.5 | N/A  50  55  60  65  70  75  80  85 | N/A  25.5 ± 5.1  23.7 ± 5.2  23.2 ± 5.0  22.7 ± 4.6  21.6 ± 4.4  20.4 ± 4.4  19.2 ± 4.7  18.2 ± 5.2 |
