# Supplementary material for: Association of Drug Burden Index with grip strength, timed up and go and Barthel index activities of daily living in older adults with intellectual disabilities: an observational cross-sectional study
Source: BMC Geriatr. 2019 Jun 24;19:173. doi: 10.1186/s12877-019-1190-3 (PMC6591943; doi:10.1186/s12877-019-1190-3)
Supplement: Supplementary file 4 — Male Grip Strength Comparison. A comparative table of male grip strength scores from The Intellectual Disability Supplement to the Irish Longitudinal Study on Ageing (IDS-TILDA), the Healthy ageing and intellectual disabilities study (HA-ID) and the Irish Longitudinal Study on Ageing (TILDA). (DOCX 16 kb) [file 12877_2019_1190_MOESM4_ESM.docx]

Additional file 4: Male Grip Strength Comparison

| **Age Range** | **IDS-TILDA Male**  **(n = 186)** | | **HA-ID Male**  **(n = 370)** | | **TILDA Male < 173cm** | | **TILDA Male ≥ 173cm** | |
| --- | --- | --- | --- | --- | --- | --- | --- | --- |
|  | *n* | *Mean Grip Strength (kg)* | *n* | *Mean Grip Strength (kg)* | *Age* | *Mean Grip Strength (kg)* | *Age* | *Mean Grip Strength (kg)* |
| 44 – 49  50 – 54  55 – 59  60 – 64  65 – 69  70 – 74  75 – 79  80 – 84  85 – 89* | 56  40  35  19  19  6  5  3  3 | 25.98 ± 10.4  23.83 ± 10.3  24.43 ± 10.8  28.63 ± 9.9  21.2 ± 10.5  25.5 ± 14.8  19.6 ± 5.7  18.33 ± 2.1  11.33 ± 12.9 | 0  72  92  87  57  35  22  2  3 | N/A  29.43 ± 12.23  28.78 ± 10.32  29.69 ± 10.45  29.28 ± 8.71  27.69 ± 9.82  24.86 ± 6.69  26 ± 8.49  18.33 ± 10.21 | N/A  50  55  60  65  70  75  80  85 | N/A  38.4 ± 7.5  36.4 ± 7.4  35.3 ± 7.2  34.0 ± 7.0  31.8 ± 6.7  29.2 ± 6.5  26.6 ± 6.3  23.8 ± 6.1 | N/A  50  55  60  65  70  75  80  85 | N/A  42.7 ± 7.8  40.7 ± 7.7  39.7 ± 7.6  38.4 ± 7.4  36.1 ± 7.1  33.6 ± 7.0  30.9 ± 6.8  28.2 ± 6.7 |
| *One participant was aged 90+ but was merged into previous category (85 – 89) as performed in a previous study [46] | | | | | | | | |
