# Supplementary material for: Association of Drug Burden Index with grip strength, timed up and go and Barthel index activities of daily living in older adults with intellectual disabilities: an observational cross-sectional study
Source: BMC Geriatr. 2019 Jun 24;19:173. doi: 10.1186/s12877-019-1190-3 (PMC6591943; doi:10.1186/s12877-019-1190-3)
Supplement: Supplementary file 5 — Timed Up and Go Comparison. A comparative table of timed up and go scores from The Intellectual Disability Supplement to the Irish Longitudinal Study on Ageing (IDS-TILDA) and the Irish Longitudinal Study on Ageing (TILDA). (DOCX 14 kb) [file 12877_2019_1190_MOESM5_ESM.docx]

Additional file 5: Timed Up and Go Comparison

| **Age Range** | **IDS-TILDA**  **(n = 251)** | | **TILDA Female**  **< 160cm** | | **TILDA Female**  **≥ 160cm** | | **TILDA Male**  **< 173cm** | | **TILDA Male**  **≥ 173cm** | |
| --- | --- | --- | --- | --- | --- | --- | --- | --- | --- | --- |
|  | *n* | *Mean TUG (sec)* | *Age* | *Mean TUG (sec)* | *Age* | *Mean TUG (sec)* | *Age* | *Mean TUG (sec)* | *Age* | *Mean TUG (sec)* |
| 44 – 49  50 – 54  55 – 59  60 – 64  65 – 69  70 – 74  75 – 79  80 – 84  85 – 89 | 132  100  92  58  49  19  10  6  2 | 14.7 ± 7.5  16 ± 10.0  17.6 ± 12.5  17.6 ± 12.0  17.5 ± 8.4  18.7 ± 6.7  24.4 ± 15.6  30.0 ± 16.8  14.6 ± 8.6 | N/A  50  55  60  65  70  75  80  85 | 8.1 ± 3.2  8.3 ± 2.9  8.6 ± 2.9  9.1 ± 3.4  10.0 ± 4.7  11.4 ± 7.4  13.9 ± 12.1  18.4 ± 18.9 | N/A  50  55  60  65  70  75  80  85 | 7.9 ± 1.6  8.1 ± 1.6  8.4 ± 1.7  8.8 ± 1.8  9.4 ± 2.2  10.3 ± 3.2  11.5 ± 5.8  13.3 ± 12.0 | N/A  50  55  60  65  70  75  80  85 | 7.9 ± 1.8  8.2 ± 2.1  8.6 ± 2.5  9.1 ± 3.4  10 ± 5.1  11.5 ± 8.1  14.1 ± 12.6  18.4 ± 18.9 | N/A  50  55  60  65  70  75  80  85 | 7.8 ± 1.3  8 ± 1.4  8.3 ± 1.6  8.8 ± 1.8  9.4 ± 2.3  10.3 ± 3.5  11.6 ± 6.2  13.3 ± 11.9 |
